# Supplementary material for: The interactome of intact mitochondria by cross-linking mass spectrometry provides evidence for coexisting respiratory supercomplexes
Source: Mol Cell Proteomics. 2017 Dec 8;17(2):216–32. doi: 10.1074/mcp.RA117.000470 (PMC5795388; doi:10.1074/mcp.RA117.000470)
Supplement: Supplemental Data [file supp_RA117.000470_133922_0_supp_23978_fzffwf.zip › spectra_annotation/mito_spectra_annotation/366-1-3-1-31-1.pdf]

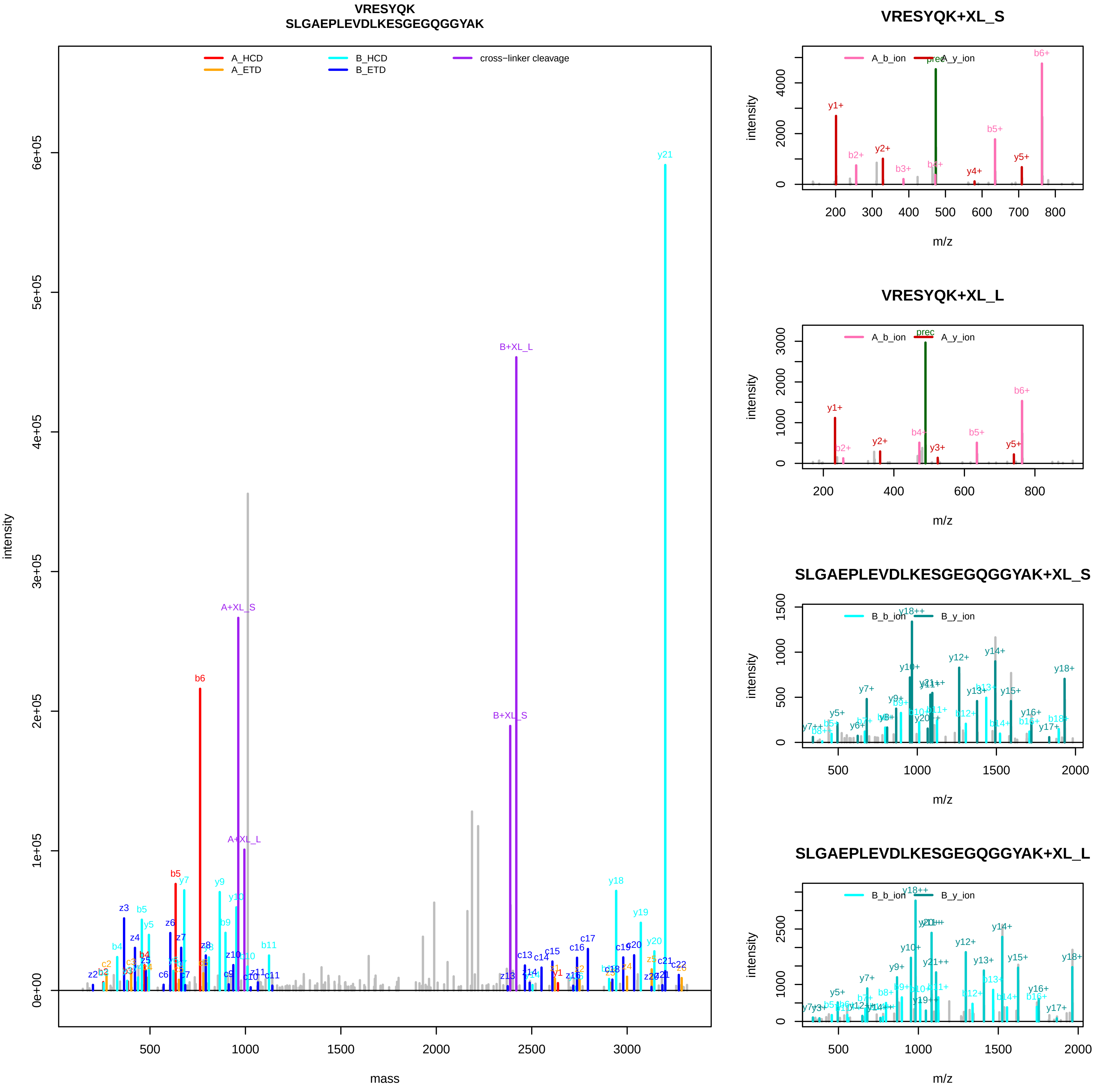

VRESYQK
SLGAEPLEVDLKESGEGQGGYAK
A_HCD
A_ETD
B_HCD
B_ETD
cross−linker cleavage
intensity
mass
0e+00
1e+05
2e+05
3e+05
4e+05
5e+05
6e+05
500
1000
1500
2000
2500
3000
y21
B+XL_L
A+XL_S
B+XL_S
b6
A+XL_L
b5
y7
y9
y18
y19
y10
z3
b5
y5
z6
z7
z4
b4
b11
c17
c16
c19
c20
c15
c14
c13
y20
c21
c22
z13
VRESYQK+XL_S
A_b_ion
A_y_ion
prec
y1+
b2+
y2+
b3+
b4+
y4+
b5+
y5+
b6+
m/z
200
300
400
500
600
700
800
0
2000
4000
VRESYQK+XL_L
y3+
0
1000
2000
3000
SLGAEPLEVDLKESGEGQGGYAK+XL_S
B_b_ion
B_y_ion
y18++
y10+
y12+
y14+
y18+
y13+
y15+
y7+
y16+
y17+
0
500
1000
1500
2000
SLGAEPLEVDLKESGEGQGGYAK+XL_L
y21++
y21+
2500
